# Aging and the Change in Fatigue and Sleep – A Longitudinal Study Across 8 Years in Three Age Groups

**DOI:** 10.3389/fpsyg.2018.00234

**Published:** 2018-03-08

**Authors:** Torbjörn Åkerstedt, Andrea Discacciati, Anna Miley-Åkerstedt, Hugo Westerlund

**Affiliations:** ^1^Department of Clinical Neuroscience, Karolinska Institute, Solna, Sweden; ^2^Stress Research Institute, Stockholm University, Stockholm, Sweden; ^3^Department of Environmental Medicine, Karolinska Institute, Solna, Sweden; ^4^Karolinska University Hospital, Stockholm, Sweden

**Keywords:** restorative sleep, sleep quality, sleep duration, aging, longitudinal

## Abstract

Fatigue is prevalent in the population and usually linked to sleep problems, and both are related to age. However, previous studies have been cross-sectional. The purpose of the present study was to investigate the trajectories of sleep and fatigue across 8 years of aging in a large group (*N* > 8.000) of individuals. A second purpose was to investigate whether fatigue trajectories would differ between age groups, and whether different trajectories of fatigue would be reflected in a corresponding difference in trajectories for sleep variables. Results from mixed model analyses showed that fatigue decreased across 8 years in all age groups, while sleep problems increased, non-restorative sleep decreased, weekend sleep duration decreased, and weekday sleep duration showed different patterns depending on age. Furthermore, the larger the decrease in fatigue, the larger was the increase in sleep duration across years, the lower was the increase of sleep problems, and the larger was the decrease of non-restorative sleep. The results suggest that aging has positive effects on fatigue and sleep and that these changes are linked.

## Introduction

Sleep disturbances is a public health problem, with about 1/3 of the population affected and with 10% seriously affected (Leger and Bayon, 2010; SBU, 2010). Impaired sleep is a major contributor to fatigue (Avlund, 2010), depression (Staner, 2010), diabetes II, cardiovascular disease, and mortality (Buysse et al., 2010), as well as to a high utilization of health resources (Kuppermann et al., 1995) and sickness absence (Lallukka et al., 2013). The link appears to be impaired biological restoration in terms of reduced brain energy levels, reduction of anabolic hormones, and impairment of immune function and insulin efficiency, etc. (Buysse et al., 2010).

Fatigue is usually seen as an inability to muster sufficient energy to carry out a task, or as a depletion of resources necessary for a task (Davies and Parasuraman, 1982). It is correlated with sleepiness but is not identical with that concept (Hossain et al., 2005). Fatigue is one of the most common medical symptoms (Wessely et al., 1997; Chalder et al., 2010). It is a central characteristic in the chronic fatigue syndrome (Sullivan et al., 2005), in burnout (Maslach and Leiter, 2005), in insomnia (Lichstein et al., 1997), as well as in health care consumption (Watt et al., 2000), and plays a major role in sickness absence (Janssen et al., 2003; Akerstedt et al., 2007) and self-care ability (Visser and Smets, 1998).

Both sleep and fatigue appear influenced by aging. Thus, sleep problems increase with age (Mallon et al., 2014) and sleep duration decreases (Ohayon et al., 2004), while fatigue increases (Schwarz et al., 2003; Avlund, 2010). This change is logical in view of the relation between sleep and fatigue. Considering the importance of age for sleep and fatigue, it is interesting that only cross-sectional studies are available, except for one study showing increased fatigue across 14 years (Westerlund et al., 2010). The problem with the cross-sectional studies is that they involve “cohort effects,” that is, there is likely to exist different life experiences in different cross-sectional age groups and life expectancy has risen substantially. In addition, one needs to be able to follow individuals across time to understand developments and to be able to relate changes in one variable to those in another variable. We expect that the steepness of the age related changes in fatigue, sleep, and perhaps stress will be an important predictor of negative outcomes with increasing age. Possibly, different age groups may have different trajectories, and retirement occurs for one group during the period investigated, which may affect the rate of change. Based on previous work increased sleep problems and fatigue should be expected. On the other hand, we have recently started to suspect that aging, in itself, may show *reductions* in fatigue if work load is adjusted for. This is based on rather small laboratory studies (Lowden et al., 2009; Dijk et al., 2010). However, if the latter studies are correct, completely different trajectories from the expected ones may be expected, that is, we might actually obtain *decreasing* fatigue and sleep problems across aging.

The purpose of the present study was to investigate the trajectories (slopes) of sleep and fatigue across 8 years of aging in a large group (*N* > 8.000) of individuals. A second purpose was to investigate whether fatigue trajectories would differ between age groups, and whether different trajectories of fatigue would be reflected in a corresponding difference in trajectories for sleep, sleep duration, sleep quality, or non-restorative sleep.

## Materials and Methods

### Design and Participants

The study was based on the Swedish Longitudinal Occupational Survey of Health, SLOSH. It is a nationally representative longitudinal study with follow-up every second year (from 2006). It has its origins in the Swedish Work Environment Survey (SWES^1^), which in turn is based on nationally representative samples of the working population. The Regional Research Ethics Board in Stockholm approved the study.

In the present paper we use data from waves 1 to 5 (labeled T0–T4) with 8159 participants. Respondents who did not complete all questions in the sleep quality index (*n* = 147) were excluded. The sample at baseline contained 43.2% males; 33.9% 18–42 year olds, 42.1% 43–56 year olds, and 24.1% 57–68 year olds; 55.3% married; 53.4% with children (living at home); 80.2% in very or rather good health. Mean age ± SD was 47.6 ± 11.6 years; 3.5% were not working.

#### Variables

Information regarding gender, age and socioeconomic position (SEP – blue-collar worker, white-collar workers, and managers), were derived from national register data at baseline (T1). Sleep duration during weekdays and weekends/days off was obtained from a question asking: “what time do you normally turn out the light” and “at what time do you normally rise.” The difference was used to represent sleep duration. Weekday and weekend sleep were correlated *r* = 0.37 for the 3^rd^ (middle) wave, with a range from *r* = 0.33 to 0.39 for the five waves (*p* < 0.001).

The 14 item Karolinska Sleep Questionnaire (KSQ) was used to assess *sleep problems* (disturbed sleep) (Åkerstedt et al., 2002, 2008, 2012; Nordin et al., 2013). The scale differentiates insomniac patients from healthy individuals (Åkerstedt et al., 2008) and correlates *r* > 0.40 with perceived stress, anxiety, depression, and burnout (Nordin et al., 2013). The items included are: difficulties falling asleep, restless sleep, repeated awakenings, and premature awakening. The responses ranged from “never” to “most days of the week” (with values from 1 to 6 assigned). Cronbach’s alpha was 0.84 at time point 3 (T3) in the present study. The index *non-restorative sleep* (Nordin et al., 2013) was constructed from the items “difficulties awakening” and “enough sleep,” not well-rested on awakening,” all scored 1–6. Chronbach’s alpha at T3 (middle wave) was 0.73. Occupation used the classification of occupations (SSYK) to produce the categories blue-collar workers, white-collar workers, and managers.

Ratings of fatigue were obtained from a single item phrased as “to what extent have you been suffering from sluggishness or lack of energy,” with response alternatives from 1 (not at all) to 5 (very much). It is significantly correlated (*r* = 0.60, *p* < 0.001) with persistent fatigue and mental fatigue (*r* = 0.57, *p* < 0.001) at T3, introduced into the cohort in wave 3. The fatigue items have been used in, for example, Ekstedt colleague’s study of burnout (Ekstedt et al., 2009).

### Statistical Analysis

The purpose of the main analysis was to identify and describe the trajectories across time for different age groups. This could be done through computation of individual regressions across time for different age groups. A more comprehensive approach, however is a multilevel approach, using mixed model regression (Rabe-Hesketh and Skrondal, 2005; Bolger and Laurenceau, 2013), which considers level 1 (time) and 2 (age groups) simultaneously. This allows individual intercepts and slopes, as well as missing data, that is, not all data points are required for a successful analysis Three groups were used in level 2, age 18–42, 43–56, and 57–68 years at T1. The choice of groups was based on the need to have one group that would retire sometime between the first wave and before the last wave, in order to have all retirees in one group. The upper limit of the oldest group was extended to 68 years since everyone worked at T1. The remainder of the sample was divided into two approximately equal groups. The analyses were adjusted for occupation and gender.

In a second analysis the trajectory of fatigue across 8 years was divided into four different groups, those who increased in fatigue across time (slope > 0), those who were close to level across time, but with a small decrease (0 to -0.021), those who had a clear decrease (-0.021 to -0.029), and those with a strong decrease (<0.029). The latter would correspond to a decrease of 0.3 fatigue units across 10 years or a decrease of 1.2 units from the age of 20 to 60 years of age on a scale from 1 to 5 (no – very high fatigue). The differences between the slopes were tested using a one-factor ANOVA. For the four groups with different fatigue slopes, the slopes for sleep duration, sleep quality and non-restorative sleep were compared using one-factor ANOVAs.

## Results

### 8-Year Trajectories for 3 Age Groups

**FIGURE 1:**
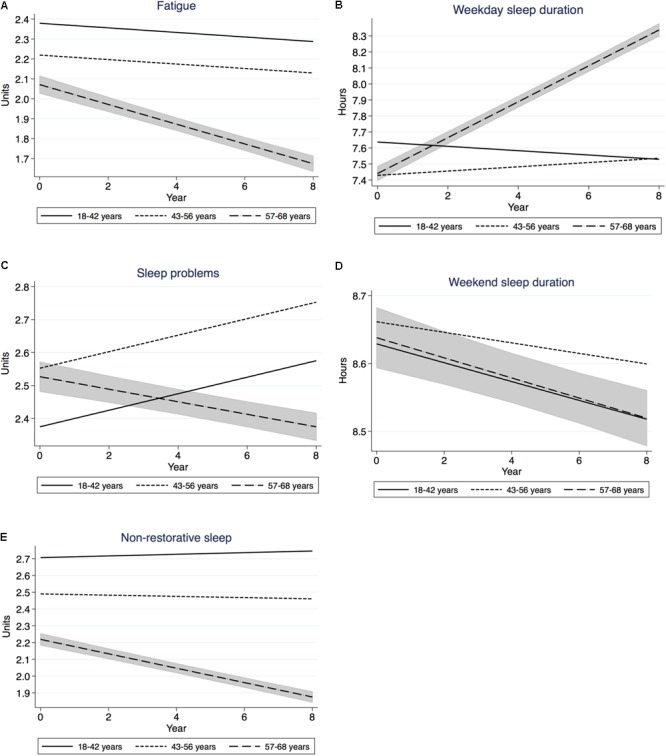
**(A–E)** Trajectories across 8 years for the main variables in three age groups. 95% Confidence interval for the oldest group. Mixed model regression. Note difference in scale for sleep duration variables.

**Figure 1A** and **Table 1** shows that fatigue decreased significantly across 8 years of follow-up. The decrease was significant for all age groups, but was most pronounced for the oldest group. The slopes of the age groups differed significantly and so did the intercepts. Thus, the oldest group had both the lowest level of fatigue at outset (intercept), and the steepest decrease across time. Correspondingly, the youngest group had the highest level of fatigue at outset, and showed the least decrease across the 8 years.

**Table 1:** Development across 8 years of fatigue, sleep duration, and sleep quality in different age strata.

	Coefficient/slope Mean ± SE Chi2	*Z*	*p*-Value for slope > 0	95% CI	constant Mean ± SE
**Fatigue 1–5 (max)**	–0.023 ± 0.002	–11.9	0.000	–0.026; -0.019	2.236 ± 0.013
18–42 years	–0.011 ± 0.004	–3.2	0.002	–0.019; -0.004	2.379 ± 0.023
43–56 years	–0.011 ± 0.003	–3.8	0.000	–0.017; -0.005	2.219 ± 0.020
57–68 years	–0.050 ± 0.093	–14.9	0.000	–0.056; -0.043	2.071 ± 0.022
Chi2/*p*-value across strata	80/0.000				345/0.000
**Sleep problems 1–5 (max)**	0.013 ± 0.002	8.4	0.000	0.010; 0.016	2.489 ± 0.002
18–42 years	0.025 ± 0.003	8.8	0.000	0.020; 0.031	2.375 ± 0.020
43–56 years	0.025 ± 0.002	10.8	0.000	0.020; 0.030	2.553 ± 0.020
57–68 years	–0.019 ± 0.003	6.9	0.000	–0.024; -0.014	2.527 ± 0.023
Chi2/*p*-value across strata	171/0.000				89/0.000
**Sleep duration Weekdays (h)**	0.033 ± 0.001	29.1	0.000	0.030; 0.036	7.49 ± 0.011
18–42 years	–0.014 ± 0.002	5.5	0.000	–0.018; -0.009	7.637 ± 0.018
43–56 years	0.013 ± 0.002	6.8	0.000	0.009; 0.017	7.429 ± 0.015
57–68 years	0.112 ± 0.003	37.6	0.000	0.106; 0.118	7.440 ± 0.022
Chi2/*p*-value across strata	1338/0.000				463/0.000
**Sleep duration Weekends (h)**	–0.012 ± 0.002	7.3	0.000	–0.015; -0.009	8.645 ± 0.012
18–42 years	–0.014 ± 0.003	–4.4	0.000	–0.020; -0.008	8.629 ± 0.021
43–56 years	–0.008 ± 0.002	3.2	0.001	–0.012; -0.033	8.661 ± 0.018
57–58 years	–0.015 ± 0.003	5.2	0.000	–0.020; -0.009	8.638 ± 0.023
Chi2/*p*-value across strata	4/0.111				11/0.004
**Non-restorative sleep**	–0.013 ± 0.001	9.6	0.000	–0.015; -0.010	2.495 ± 0.011
18–42 years	0.005 ± 0.003	1.9	0.059	–0.000; 0.010	2.707 ± 0.020
43–56 years	–0.004 ± 0.002	1.9	0.058	–0.007; 0.000	2.490 ± 0.016
57–68 years	–0.043 ± 0.002	18.8	0.000	–0.047; -0.038	2.219 ± 0.018
Chi2/*p*-value across strata	227/0.000				872/0.000

*Results from mixed model regression.*

*N = 2733, 3312, 2130 for the youngest, intermediate, and oldest age groups, respectively, with only marginal variations between age groups. The percentage retired was 0% for year 1 and 18.3% for year 8. The percentage not working was 3.5% for year 0 and 25.6% for year 8. Chi2 represents the test for significance for the differences across age groups for slopes and constants.*

Sleep problems showed a significant overall increase and significant changes for all age groups (**Figure 1** and **Table 1**), but the change in the oldest group was one of a decrease, while the two younger groups increased. The difference in slopes between age groups was significant, as was the difference in intercept. Thus, the youngest group had the lowest level of sleep problems at outset but increased across time, whereas the highest group had a high level (but not the highest), but decreased across time. Across 10 years the oldest group would have decreased 0.19 units and the youngest group would have increased 0.25 units. Thus, the older group would have shown less sleep problems than the younger group after 10 years.

Sleep Duration on weekdays increased significantly for all participants and for the oldest two groups, but the youngest group showed a significant decrease (**Figure 1** and **Table 1**). The difference in slopes was significant, and so was the difference in intercept. The result indicates that the older group had the shortest sleep at outset, but a steep (but not the steepest) increase across time. Across 10 years this corresponds to +0.11 h. The youngest group showed the longest sleep at outset, but a decline across years. Across 10 years this corresponds to -0.14 h. This means that the older group would have surpassed the youngest group in weekday sleep duration after 10 years.

Sleep Duration on weekends showed a significant overall decrease, as well as a decrease for each age group (**Figure 1** and **Table 1**). The difference in slopes was not significant. The difference in intercept was significant. Thus, all age groups showed a uniform decrease.

Non-restorative sleep showed a significant overall decrease, as did the oldest group (**Figure 1** and **Table 1**). The difference in slopes was significant, as was the difference in intercepts. This means that the oldest group showed a particularly strong decrease, and from a particularly low level of problems. The youngest group, instead, did not increase significantly but showed a high level of non-restorative sleep.

### 8-Year Sleep Trajectories for Groups With Different Fatigue Slopes

**Table 2** presents the slope across 8 years for four sleep variables according to the direction and size of the slope of fatigue. *Weekday sleep duration* showed significantly different slopes, with increasing steepness across the four groups, with the strongest increase in weekday sleep duration for individuals with the steepest decrease in fatigue. The weakest increase in weekday sleep duration was seen for the group with increasing fatigue. The intercepts showed significant differences without a clear pattern.

**Table 2:** Coefficients for slopes 95% Confidence Interval and constant for sleep variables in groups with different degrees of change in fatigue across time.

Slopes of fatigue	Mean level Coefficient ± SE and Chi2/*p*	*Z*	*p*	95% CI	Constant ± SE and Chi2/*p*
**Weekday sleep duration**					
>0	0.021 ± 0.008	2.6	0.011	0.005; 0.037	7.75 ± 0.045
–0.021, 0	0.023 ± 0.003	8.8	0.000	0.018; 0,028	7.64 ± 0.015
–0.029, -0.21	0.030 ± 0.003	12.0	0.025	0.025; 0.035	7.60 ± 0.015
< -0.29	0.047 ± 0.002	19.1	0.000	0.042; 0.052	7.64 ± 0.015
Chi2/*p*-value across strata	54/0.000				14/0.002
**Weekend sleep duration**					
>0	–0.011 ± 0.008	1.3	0.186	–0.027; 0.005	8.79 ± 0.050
–0.21, 0	–0.010 ± 0.003	3.3	0.001	–0.015; -0,004	8.65 ± 0.017
–0.029, -0.22	–0.013 ± 0.003	4.3	0.000	–0.018; -0.007	8.57 ± 0.017
< -0.29	–0.013 ± 0.003	5.1	0.000	–0.018; -0.008	8.55 ± 0.016
Chi2/*p*-value across strata	1/0.850				20/0.000
**Sleep problems**					
>0	0.077 ± 0.008	9.1	0.000	0.060; 0.093	3.23 ± 0.058
–0.21, 0	0.041 ± 0.003	14.5	0.000	0.036; 0.047	2.87 ± 0.018
–0.029, -0.22	0.008 ± 0.003	3.0	0.003	0.003; 0.013	2.45 ± 0.016
< -0.29	–0.019 ± 0.002	8.0	0.000	–0.023; -0.014	2.19 ± 0.015
Chi2/*p*-value across strata	361/0.000				1036/0.000
**Non-restorative sleep**					
>0	0.066 ± 0.007	8.9	0.000	0.052; 0.081	3.26 ± 0.053
–0.21, 0	0.009 ± 0.002	3.8	0.000	0.004; 0.014	2.80 ± 0.016
–0.029, -0.22	–0.017 ± 0.002	7.2	0.000	–0.021; -0.012	2.39 ± 0.014
< -0.29	–0.040 ± 0.002	20.3	0.000	–0.044; -0.037	2.04 ± 0.013
Chi2/*p*-value across strata	438/0.000				1364/0.000

*N = 327, 2616, 2616, 2600 for the four groups, with marginal variations between variables. Total *N* = 8159. CI, confidence interval.*

*Weekend sleep duration* showed significant decreases for all four groups, but no significant differences between groups. The intercepts differed significantly, with lowest weekend sleep duration for the group with the steepest decline.

*Sleep problems* showed a significant difference across groups, with the increasing slope for sleep problems becoming less steep slopes moved from >0 to <0.029, in which latter group the slope became significantly negative. Also the intercept decreased strongly. Thus, the group with the steepest decrease of fatigue had the lowest sleep problems at outset and also the steepest decrease of sleep problems. In contrast, the group that showed increasing fatigue across the 8 years had the highest level of sleep problems at outset, as well as an increasing trajectory across time.

*Non-restorative sleep* showed a significant difference of slopes. From an increase across 8 years in the group with a positive fatigue slope, it changed to a decrease in slope for those having the steepest decrease in fatigue across time. The intercept also decreased from a high level of no-restorative sleep in the group with a positive fatigue slope, to a lowest level of non-restorative sleep among those who had the steepest decrease in fatigue across time. Thus, those who had a strong decrease in fatigue also had a strong decrease in non-restorative sleep – at an already low level at the outset. The group with the increasing slope of fatigue also had a strong increase in non-restorative sleep – at a high level of non-restorative sleep at outset.

**Table 3** presents the number of participants in the groups with different slopes.

**Table 3:** Gender and age for groups with different slopes of fatigue.

Slopes of fatigue	*N*	Males (%)	19–42%	43–56%	57–68%	Working (%)
>0	327	72.8	40.7	11.2	11.2	56.2
–0.021, 0	2616	60.8	40.1	17.3	17.3	45.0
–.029, -0.21	2616	56.3	36.1	22.2	22.2	41.3
< -0.29	2699	50.0	24.2	37.8	37.8	45.6

### Retirement

Since the oldest age group included those who had retired sometime between the first and last point of measurement we also analyzed the difference from the year before to the year after this event. The mixed model analysis showed that most variables improved, that is complaints and weekday sleep duration increased. This was the case for fatigue (β = -0.27 ± 0.03, *t* = 8.6, *p* < 0.000), sleep problems (β = -0.14 ± 0.04, *t* = 3.4, *p* < 0.000), non-restorative sleep (β = -0.25 ± 0.03, *t* = 8.1, *p* < 0.000), weekday (WD)sleep duration (β = 0.52 ± 0.04, *t* = 12.5, *p* < 0.000), and sleep duration WE (β = 0.03 ± 0.04, *t* = 0.07, *p* = 0.946).

## Discussion

The decrease in fatigue across time in different age groups and the lower level of fatigue in the old group at outset, constitute strong evidence for a reduction of fatigue with age. There seem to be no previous studies of longitudinal changes in fatigue, except for the finding of a 10-year *increase* in fatigue toward retirement (Westerlund et al., 2010). The latter study involved employees at one particular (utility) company and the outcome could, possibly, be due to increases in workload in that particular organization, but this is speculative. It also used the terms “mental fatigue” and “physical fatigue” which may differ somewhat from the term used in this paper. The present results are also at odds with findings of higher fatigue in higher age groups (Schwarz et al., 2003; Avlund, 2010). However, there is a risk that cross-sectional studies may suffer from cohort effects, with different age groups having been exposed to different amounts of education, work stress, and other factors. A longitudinal approach, such as in the present study avoids this problem. The results of the present longitudinal study do however, agree with the finding of Dijk et al. (2010) that sleepiness (subjective and objective) is lower in older participants or that subjective sleepiness and long eyeblink duration are both lower during simulator driving in older participants (Lowden et al., 2009; Dijk et al., 2010). Fatigue and sleepiness are not the same thing but is usually highly correlated across time (Åkerstedt et al., 2014). The observation of decreasing fatigue across aging needs further corroboration, however. And, clearly, the reduction of fatigue in the oldest group clearly included a strong retirement effect.

The second major finding was the decrease in weekend sleep duration (WE) across time in all age groups. There is no previous longitudinal or cross-sectional data to compare with, but polysomnographic studies, which often provide a longer time in bed (around 8 h) than sleep during the working week, show decreasing sleep with increasing age (Ohayon et al., 2004). Since weekend sleep is less restricted by work and other obligations, it may provide compensatory sleep after a working week with curtailed sleep. Possibly, a sleep opportunity with reduced curtailment, such as weekend sleep, may also reflect a need for sleep, even if this concept is rather elusive and lacks definition. Experimental extension of the time in bed to 16 h seems, after some days of variability to become stable at a duration considerably above the habitual working week sleep duration (Klerman and Dijk, 2008). Interestingly, 70 year olds could “produce” 7.4 h of sleep on average, whereas 25 year olds could produce 8.9 h. However, increasing age also means reduced SWS [deep sleep = stage N3 (earlier label = Stage 3+4)], increased, N1 (superficial sleep = earlier label = Stage 1), increased awakenings (Schwarz et al., 2017), all of which suggest poor sleep. This topic of aging and objective/subjective sleep quality needs further research. As expected, retirement did not affect weekend sleep.

In contrast, weekday sleep, in the present study, showed a very different pattern with little change across years except in the oldest group. We assume that the younger groups had to curtail their sleep because of work hours, but the present cohort lacks this kind of data. The older group includes a majority of individuals that retired during the 8 years studied. They were thus able to extend their sleep during the conventional weekdays as demonstrated in the retirement analysis. If weekend sleep, at least partly, represents the need for sleep, it is obvious that particularly younger individuals suffer a sleep deficit during most days of the week.

Non-restorative sleep also decreased with aging in all age groups, and was lower at outset in the oldest group. This pattern has been seen in cross-sectional studies (Wakasugi et al., 2014) and seems logical, considering the increased tendency to wake up early with increasing age. Non-restorative sleep combines ease of awakening, being well-rested, and getting sufficient sleep, all of which seem to be logical effects of increasing earliness. A decrease of non-restorative sleep would also fit in with the decrease in weekend sleep duration (in all age groups), which could reflect a decrease of the need for sleep. And, again, retirement accounted for part of the aging effect in the older group.

Finally, sleep problems increased with aging, but not in the oldest group, where a decreasing trajectory was seen. The overall pattern of increase agrees with much previous research (Mallon et al., 2014), but the deviant pattern of the older group could perhaps again be related to the positive effect of retirement, which may have counteracted the general age trend toward worse sleep. As demonstrated by Vahtera et al. (2009) retirement has a positive effect on sleep disturbances.

Another important question concerned whether individuals with strongly decreasing fatigue or weakly decreasing or increasing fatigue would show corresponding patterns for sleep variables. This was not borne out for weekend sleep duration, but to some extent for weekday sleep. Thus, the steeper the fall in fatigue, the steeper was the increase in sleep duration. This is not to infer causality, although the results do not contradict a causative relation, where increasing trajectories of weekday sleep are associated with decreasing trajectories of fatigue.

With respect to sleep problems and non-restorative sleep, the pattern was the same. Thus, with increasing steepness of the decrease of fatigue, both sleep problems and non-restorative sleep showed a change from slightly increasing, to steeply decreasing slopes. This was also combined with a change from high problem levels to very low levels at the outset. Taken together, the results indicate that when sleep problems or non-restorative sleep decreases, also fatigue decreases. The present authors are not aware of similar work in the literature, so the observations need corroboration and, again, no conclusions about causation can be drawn. However, the overall impression is one of a strong improvement in fatigue with aging and a corresponding improvement in sleep.

The pattern of decreased fatigue with no change in weekday sleep duration (for the younger and still working individuals), together with a reduction in weekend sleep, constitutes a paradox with respect to the expectation that decreased fatigue should be associated with increased sleep duration. Apparently, aging changes this association. The reason is not clear, but sleepiness decreases with increasing age (Dijk et al., 2010), which could mean that the need for sleep decreases with age. There is no empirical data to support this hypothesis, but it is a testable.

The present study has several limitations. One concerns the single item fatigue variable. However, it correlated well with specific fatigue items (*r* > 0.56) introduced in later waves. A second limitation is the reliance on only subjective data. Still, objective data are not used in the diagnosis of insomnia (Iber et al., 2007), or when estimating sleep problems in the general population.

Taken together, the age related decrease in fatigue, weekend sleep duration, and non-restorative sleep, as well as the decrease in sleep problems in the oldest group suggest that aging, at least in relatively healthy individuals, seems to have positive effects on fatigue and sleep. This runs counter to much previous research based on cross-sectional cohorts. Since the findings on age-trajectories of sleep and fatigue lack previous studies, there is a need for corroboration from similar self-report approaches, but also from actigraphy or polysomnography.

## Author Contributions

TÅ provided the idea and wrote the manuscript. AD made the statistical analyses and commented on the manuscript. HW provided the cohort and commented on the manuscript. AM-Å did part of the analyses and commented on the manuscript.

## Conflict of Interest Statement

The study was funded by the Research Foundation of the AFA Insurance Company. The research foundation is separate from the insurance part of the company. The funder was in no way involved in the study design, data collection or interpretation of the data. All the authors declare that the research was conducted in the absence of any commercial or financial relationships that could be construed as a potential conflict of interest.

## Footnotes

1 www.scb.se
